# Reprogramming of a defense signaling pathway in rough lemon and sweet orange is a critical element of the early response to ‘*Candidatus* Liberibacter asiaticus’

**DOI:** 10.1038/hortres.2017.63

**Published:** 2017-11-29

**Authors:** Qibin Yu, Chunxian Chen, Dongliang Du, Ming Huang, Jiqiang Yao, Fahong Yu, Ronald H Brlansky, Frederick G. Gmitter

**Affiliations:** 1 University of Florida, Institute of Food and Agricultural Sciences, Citrus Research and Education Center, Lake Alfred, FL 33850, USA; 2 USDA, ARS, SEFTNRL, 21 Dunbar Road, Byron, GA 31008, USA; 3 Interdisciplinary Center for Biotechnology Research, University of Florida, 2033 Mowry Road, Gainesville, Florida 32611, USA

## Abstract

Huanglongbing (HLB) in citrus infected by *Candidatus* Liberibacter asiaticus (*C*Las) has caused tremendous losses to the citrus industry. No resistant genotypes have been identified in citrus species or close relatives. Among citrus varieties, rough lemon (*Citrus jambhiri*) has been considered tolerant due to its ability to produce a healthy flush of new growth after infection. The difference between tolerance and susceptibility is often defined by the speed and intensity of a plant’s response to a pathogen, especially early defense responses. RNA-seq data were collected from three biological replicates of *C*Las- and mock-inoculated rough lemon and sweet orange at week 0 and 7 following infection. Functional analysis of the differentially expressed genes (DEGs) indicated that genes involved in the mitogen activated protein kinase (MAPK) signaling pathway were highly upregulated in rough lemon. MAPK induces the transcription of WRKY and other transcription factors which potentially turn on multiple defense-related genes. A Subnetwork Enrichment Analysis further revealed different patterns of regulation of several functional categories, suggesting DEGs with different functions were subjected to reprogramming. In general, the amplitude of the expression of defense-related genes is much greater in rough lemon than in sweet orange. A quantitative disease resistance response may contribute to the durable tolerance level to HLB observed in rough lemon.

## Introduction

Huanglongbing (HLB) or citrus greening is one of the most destructive plant diseases in the world.^1^ The disease is caused by the bacterium, *Candidatus* Liberibacter asiaticus (*C*Las), a phloem-inhabiting, α-proteobacteria, and is transmitted by the insect vector, Asian citrus psyllid (*Diaphorina citri*).^2^ It is generally recognized that after infection or inoculation, CLas bacteria migrate through the phloem and accumulate there, resulting in the formation of sieve plugs, which contribute to HLB symptoms.^3^ Since no toxins, cell wall degrading enzymes or specialized secretion systems have been identified in the *C*Las genome, it is believed that the disease symptoms are the result of host metabolic imbalances brought about by nutrient depletion or interference with nutrient transport.^4^ The relationship between plant hosts and their associated microbes is essentially guided by a plant innate immunity system. Plants have evolved two layers of immune systems, pathogen-associated (or microbe-associated) molecular patterns (PAMP)-triggered immunity (PTI) and effector-triggered immunity. PTI is mediated by pattern-recognition receptors (PRRs) that recognize PAMPs, whereas effector-triggered immunity is mediated by resistance (R) proteins that recognize pathogen effectors.^5^ The role of these immunity systems in citrus greening disease and the transcriptomic response of citrus is poorly understood, especially in regards to the early defense response of rough lemon to HLB infection.

No source of HLB resistance has been identified in citrus species or closely-related relatives. Folimonova *et al.*^6^ classified the response of 30 citrus genotypes to HLB from sensitive to tolerant. They classified Eureka lemon and Persian lime as tolerant, with little or no HLB symptoms and no strong correlation between bacterial titer and disease severity. Zhang *et al.*^7^ also reported that HLB-affected scions of lemon had a higher titer of *C*Las, survival rate, and pathogen transmission rate than pummelo (*Citrus maxima*) scions. *Citrus limon* Burm. F., represented by ‘true’ lemons such as ‘Eureka’, contains genome contributions from three species, (*C. maxima*, *C. reticulata*, and *C. medica*) and is distinct from rough lemon, though related.^8^ Our previous study also showed that rough lemon is tolerant of HLB.^9^ Once rough lemon trees are infected and symptomatic, they can be rejuvenated by the continued growth of new shoots with few or no foliar symptoms of the disease, and they repeat this cycle for many growing seasons. In contrast, sweet orange exhibits continuous growth inhibition and eventual dieback.^9^

Understanding the differences in response of the tolerant and susceptible citrus genotypes to HLB is essential for developing genetic strategies that can produce tolerant, or perhaps even resistant, varieties. Most of transcriptomic studies of citrus-*C*Las interactions have been conducted on different tissues (leaves, fruit and root) of sweet orange,^10–13^ and roots of tangerine;^14^ while only two studies have focused on the leaves of tolerant genotypes of rough lemon^9^ and US-897.^10^ US-897, however, is an intergeneric hybrid of *Citrus* spp. and *Poncirus* spp, and may not provide a complete understanding of HLB response in citrus varieties.^9^ Using a comparative approach for understanding plant-microbe interactions can be complicated because the response and effect of candidate genes in tolerant genotypes may differ in the genetic background of susceptible trees due to epistasis. Previous studies have indicated that resistance and susceptibility can often depend on the speed and magnitude of the defense mechanism employed and the ability of the pathogen to suppress host response.^4,15,16^ It has been reported that infection of sweet orange with *C*Las does not lead to a significant induction of defense-related genes at the early stages of the infection process.^10,11^ Therefore, it is important to learn if HLB-tolerant rough lemon recognizes the presence of *C*Las at an early stage of infection and whether or not a defense response is elicited. Leaves of citrus trees in the field can remain asymptomatic and the titer of *C*Las by reverse transcription-quantitative PCR (RT-qPCR) can remain undetectable as long as two years after the initial psyllid transmission of *C*Las due to the need for the presence of a high bacterial titer for detection.^17^ False negatives and false positives are common due to low titers of bacteria inside the phloem, the presence of PCR amplification inhibitors in the phloem sap, and non-specific amplification.^18^ *C*Las in greenhouse-inoculated plants can remain undetectable for 5–9 weeks ^10^ or 20 weeks.^9^ In a time course study of *C*Las infection, Fan *et al.*^9^ reported that a greater number of stress response genes were distinctively modulated in rough lemon than in sweet orange. The study, however, did not identify any genes at a significant false-discovery rate (FDR) rate in the early stages (5 weeks) of the infection. In another study, no significant induction of defense-related genes was observed in sweet orange inoculated to *C*Las over a 5- to 9-week period of time,^10^. Both of the mentioned studies utilized an Affymetrix citrus microarray for their transcriptome analysis. However, it is not known how many unique citrus genes are actually represented in the chip. Misleading interpretations of microarray results can also occur due to non-specific hybridization.^14^ Next-generation RNA sequencing technology, however, can reveal rare and unknown transcripts, thus offering a more precise and accurate picture of the transcriptome.^13,19^

In the present study, a whole-genome transcriptional analysis of rough lemon and sweet orange leaves was conducted at 7 weeks post-inoculation in order to identify genes that were induced as part of an early response to *C*Las. Leaf samples collected immediately after inoculation (week 0) were used as a baseline, which was not done in previous transcriptome studies of *C*Las in citrus.^4,10,11^ By using week 0 as a baseline, the natural variation among biological replications could be minimized and the number of genes whose expression was significantly affected by *C*Las could be maximized. The response of mock-inoculated and *C*Las-inoculated rough lemon and sweet orange were separately analyzed and the two datasets were subsequently compared, in order to identify biological mechanisms associated with each genotype and infection versus mock-inoculation. The transcriptome analysis identified statistically significant differentially expressed genes (DEGs) between rough lemon and sweet orange in response to *C*Las. A distinct difference in the defense response between rough lemon and sweet orange in *C*Las-inoculated leaves was observed. Not only were two distinct sets of DEGs identified but greater amplitude in the profile of defense response genes in rough lemon, relative to sweet orange, was also observed. The present study provides a comprehensive overview of the early transcriptional reprogramming that occurs in rough lemon in response to *C*Las.

## Materials and methods

### Plant material and experimental design

**Figure 1:**
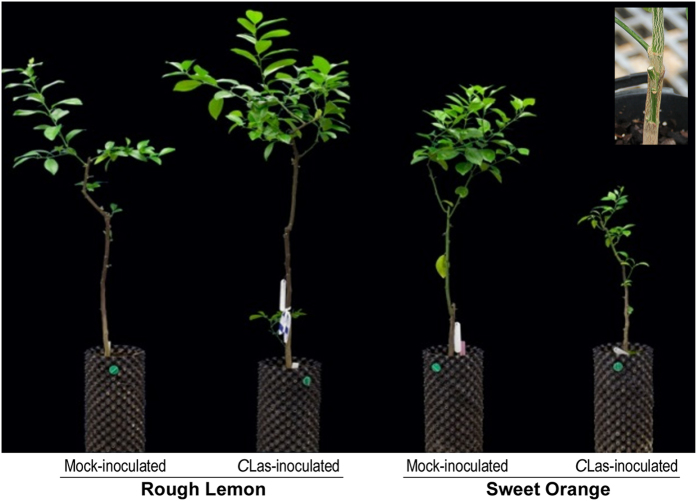
Phenotype of sweet orange and rough lemon trees 18 months after *C*Las-inoculation.

Young trees were inoculated as described by Fan *et al.*^9^ Briefly, two-year-old seedlings of rough lemon (*C. jambhiri* Lush.) and ‘Madam Vinous’ sweet orange (*C. sinensis* L Osb.) were graft-inoculated with 3–4 cm bud wood from *C*Las-infected Carrizo citrange (*C. sinensis×P. trifoliata* L. Raf.) trees maintained under greenhouse conditions in order to provide an inoculum source for various HLB-related experiments; Carrizo was used as the inoculum source because it is immune to citrus tristeza virus (CTV), a common contaminant in field source trees of *C*Las. Control seedlings were grafted with 3–4 cm budwood from healthy Carrizo trees. All these plants were kept in a United States Department of Agriculture Animal and Plant Health Inspection Service and Center for Disease Control-approved and secured greenhouse at the University of Florida, Citrus Research and Education Center, Lake Alfred. Leaf samples were collected from the middle of the plant crown and approximately 60–80 cm from the inoculation site. Three biological replicates were produced for each species in each treatment (Figure 1). Reverse transcription-quantitative PCR (RT-qPCR) was performed to confirm the presence of *C*Las in the inoculum source and in inoculated plants as previously described.^8^ Four fully expanded leaves were sampled separately from *C*Las-inoculated plants and mock-inoculated plants (used as controls) of each species at 0 and 7 weeks after inoculation. Leaves were immediately frozen in liquid nitrogen and stored at −80 °C until further use. Three biological replicates were analyzed for each condition (Figure 1). In total, 12 plants and 24 leaf samples were collected (2 species×2 treatments×3 replications×2 time points). Selection of week 7 as the time point representing early response to *C*Las was based on results of a preliminary pilot experiment conducted to determine the time point at which the maximum number of DEGs changing in expression was first initiated in response to *C*Las.

### PCR detection of *C*Las

DNA from leaf midribs and petioles was extracted using the Plant DNeasyMini Kit (Qiagen, Valencia, CA, USA) according to the manufacturer’s instructions. RT-qPCR assays were performed as previously described.^20^ Amplifications were performed in an Agilent Mx3005P (Agilent Technology) real-time PCR system using the Brilliant III Ultra-Fast QPCR Master Mix (Agilent Technology Inc, Waldbronn, Germany). Plants were considered PCR-positive when CT (cycle threshold) values were below 30.

### RNA extraction and sequencing library construction

Total RNA from the 24 samples described above was extracted using TRIzol® Reagent following the manufacturer’s protocol. RNA was further purified using the TURBO DNA-free™ kit to eliminate genomic DNA. A NanoDrop Spectrophotometer (NanoDrop Technologies, Inc., Wilmington, DE, USA) and gel electrophoresis were used to assess RNA quality and quantity. RNA quality was further assessed using an Agilent 2100 Bioanalyzer (Aglient Technologies Inc, Waldbronn, Germany). Ribosomal RNA was removed from the total RNA using a Ribo-Zero rRNA removal kit for using plant seed/plant leaf following the manufacturer’s protocol. Subsequently, 50 ng of the purified RNA from each sample were used for library construction utilizing an Epicentre ScriptSeq v2 RNA-Seq library preparation kit according to the user’s guide.

Illumina RNA library construction was performed at the Interdisciplinary Center for Biotechnology Research (ICBR) Gene Expression Core, University of Florida (UF). Briefly, rRNA-depleted RNA was fragmented by incubation at 85 °C, and then reverse transcribed using random primers containing a 5ʹ-tagging sequence. A 3ʹ-tag was added using a terminal-tagging reaction resulting in Di-tagged, single-stranded cDNA. Following purification, the di-tagged cDNA was amplified by limited-cycle PCR, in order to add the Illumina adaptor sequences. The amplified libraries were purified using Agencourt AMPure beads (Beckman Coulter, catalog # A63881). The library size and mass was assessed by analysis in the Bioanalyzer. Typically, a 200–2000 broad library peak was observed with the highest peak at ~500 bp. Quantitative PCR was used to validate the library’s functionality, using the KAPA library quantification kit (Kapa Biosystems, catalog number: KK4824). Finally, the libraries were pooled in equimolar concentration and sequenced on an Illumina 2×100 HiSeq 2000 (Illumina Inc., San Diego, CA, USA)

### RNA-seq data analysis

Raw sequencing reads were initially cleaned as follows. Ambiguous residues were trimmed off from both sides of the sequence. Bases with a Phred quality below 20 from the 3ʹ end of the sequence were removed. Reads shorter than 40 bases or those containing >10 bases with a quality rating below 20 were also discarded. In addition, reads consisting of repetitive single bases that accounted for >60% of the length at the 5ʹ or 3ʹ end were also discarded. Approximately 50–65% of reads were retained after the initial processing, providing 45–75 million clean reads for each of the 24 samples (Table 1). The *Citrus clementina* genome v1.0 (JGI) genomic sequence (http://www.citrusgenomedb.org/species/clementina/genome1.0) was used as reference genome for mapping the reads. Sequencing reads from each sample were mapped independently to the reference sequences using gmap v3 (http://research-pub.gene.com/gmap/src/gmap-gsnap-2012-07-20.v3.tar.gz).^21^ This step successfully mapped all of the cleaned reads to the genome; resulting in a total of ~56.3% of the reads that were uniquely mapped to the genome.

Gene expression values were determined as follows. The number of mapped reads for each individual gene was counted using an in house perl script. The gene annotation file in GFF format was downloaded from Phytozome v1.0 http://www.phytozome.net (Phytozome: Cclementina 182 v1.0.gene.gff3.gz). The DESeq package in R was used for gene expression analysis.^22^ DESeq uses the negative binomial distribution, with variance and mean linked by local regression, to model the null distribution of the count data. Significant up- and downregulated genes were selected using two cutoffs: an adjusted *P* value of 0.05 and a minimum fold-change of 2.0. Gene sets from each combined sample and treatment were annotated using Blast2GO[67] to assign Gene Ontology (GO) terms to each gene. Lists of transcripts that were differentially expressed using a false-discovery rate (FDR) <0.05 in the pairwise comparisons were used in the Fisher’s Exact Test in Blast2GO in order to identify GO terms that were significantly over-represented. The biological interpretation of the DEGs was further assessed by assigning the genes to metabolic pathways using the Kyoto Encyclopedia of Genes and Genomes (KEGG).^23^ Subnetwork enrichment analyses were run on the data set using Pathway Studio 10.0.^24^

### Reverse transcription-quantitative PCR

Nine DEGs that were identified by RNA-seq as either up- or downregulated in response of *C*Las were selected for validation by RT-qPCR. The RT-qPCR analyses were performed in two steps. First strand cDNA was synthesized from 0.3 μg of total RNA using an Affinityscript QPCR cDNA Synthesis Kit (Agilent Technologies), according to the manufacturer's recommendations. Primers for the twelve selected genes were designed using Primer-BLAST software at NCBI (http://www.ncbi.nlm.nih.gov/tools/primer-blast/), and are listed in Supplementary Table S1 of Supplementary File 6. RT-qPCR was performed using a Brilliant III Ultra-Fast SYBR Green QPCR Master Mix (Agilent Technologies), following the manufacturer's recommended procedures. GAPDH was used as a reference gene to normalize the expression of the other analyzed genes. PCR reactions were performed using 2 μl of cDNA, 0.25 nM of each primer, and 10 μl of 2× SYBRGreen PCR master mix (Aglient Technologies Inc, Santa Clara, CA, USA) in a 20-μl volume. A negative control was included using water as a template for each primer pair. Specificity of the amplification reactions was checked by post-amplification dissociation curves and by sequencing the reaction products. The fluorescent intensities were used to determine relative mRNAlevels with MxPro software.

## Results

### *C*Las detection and HLB symptom development

Leaf samples collected at week 7 did not exhibit the characteristic blotchy mottle appearance and were RT-qPCR negative for *C*Las. Continued RT-qPCR analysis for *C*Las was conducted every two weeks after inoculation. Positive confirmation of *C*Las in inoculated plants was not obtained in rough lemon and sweet orange until 23 weeks post inoculation. Even after 8 months, however, rough lemon did not exhibit any signs of growth inhibition, and continued growth of new shoots with few or no symptoms was observed; however, the typical blotchy mottled appearance was commonly found on mature, older leaves (Figure 1). In contrast, severe levels of blotchiness were observed on mature and older leaves of *C*Las- inoculated sweet orange and growth was significantly inhibited, with the rare production of new shoots toward the end of the experiment (Figure 1). *C*Las was not detected by RT-qPCR in samples from any of the mock-inoculated rough lemon or sweet orange throughout the entire experiment.

### Comparative RNA-seq analysis

A comparison of the RNA-seq data of the tolerant rough lemon and susceptible sweet orange was conducted using samples collected in week 7. RNA-seq data obtained at week 0 were used as a baseline. A total of 24 cDNA libraries (2 treatments×2 varieties×3 replications×2 time points) were sequenced, generating 1274.9 million reads and 740.4 million unique reads were assigned (Supplementary File 1, Supplementary Table S1). The reads were trimmed and aligned to the *Citrus clementina* genome available on the Phytozome website (http://www.phytozome.net). Expressed genes and transcript isoforms were identified and annotated using the *C. clementina* genome v.1 assembly consisting of 301.4 Mb spread over 1398 scaffolds. RNA-seq analysis was independently performed using a pair-wise comparison between week 7 and the baseline week 0 for inoculated and mock-inoculated rough lemon and sweet orange using DEseq for each treatment.^22^

**Figure 2:**
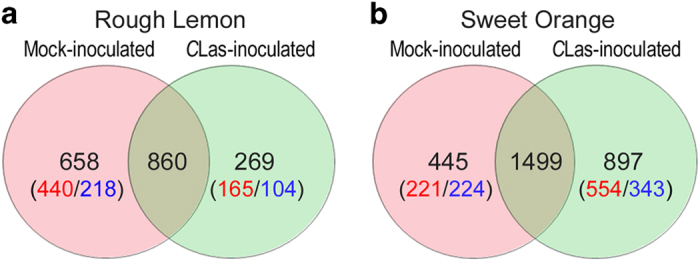
Venn diagrams of differentially expressed genes in mock- and *C*Las-inoculated rough lemon (**a**) and sweet orange (**b**). The number of significantly up-regulated genes is shown (first) in red, and significantly downregulated genes (second) in blue.

**Figure 3:**
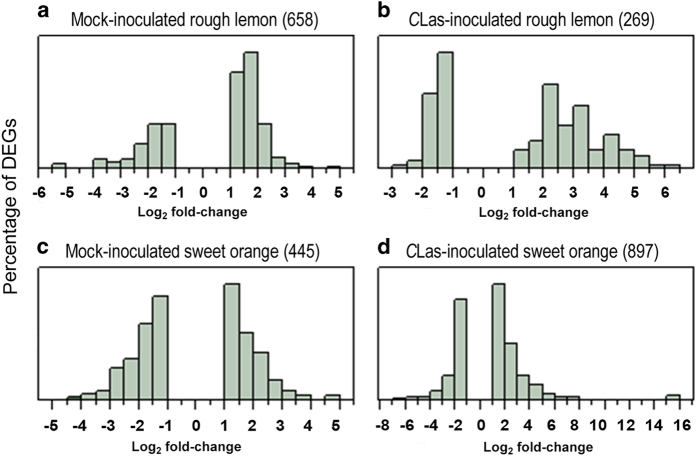
Distribution of significant differentially expressed genes (DEGs): (**a**) *C*Las-inoculated rough lemon; (**b**) Mock-inoculated rough lemon; (**c**) *C*Las-inoculated sweet orange; (**d**) Mock-inoculated sweet orange.

A total of 33 930 unique transcripts were identified and quantified but 1884 genes did not have any corresponding *Arabidopsis* orthologs (Supplementary File 2, Supplementary Table S1). Due to duplications of genes matching a single *Arabidopsis* identifier, only 14,393 *Arabidopsis* identifiers were obtained. A total of 3266 DEGs were identified in the combined inoculated and mock-inoculated rough lemon and sweet orange by a pair-wise comparison between week 7 and week 0 (Supplementary File 2, Supplementary Table S2). More specifically, 1518 and 1129 DEGs were identified in the mock-inoculated and *C*Las-inoculated samples of rough lemon, respectively. For sweet orange, 1944 and 2396 DEGs were identified in the mock-inoculated and *C*Las-inoculated samples, respectively. Further analysis indicated that 860 DEGs overlapped between the mock- and *C*Las-inoculated samples of rough lemon (Figure 2a), whereas 1499 DEGs overlapped between mock- and *C*Las-inoculated samples of sweet orange (Figure 2b). A total of 2024 DEGs were selected for further functional analysis after the removal of mock- and *C*Las-inoculated overlapping genes (Supplementary File 2, Supplementary Table S3). The removal of the overlapping genes resulted in a total of 658 DEGs and 269 DEGs for mock- and *C*Las-inoculated samples of rough lemon, respectively, and 445 DEGs and 897 DEGs for mock- and *C*Las-inoculated samples of sweet orange (Supplementary File 2, Supplementary Table S3). Among the 269 DEGs in inoculated rough lemon samples, 76 were upregulated⩾3-fold (log2), abbreviated as log-fold change (LFC), while no DEGs with this magnitude of downregulation were observed (Figure 3). *C*Las-inoculated samples of sweet orange contained 897 DEGs, of which 119 DEGs were upregulated ⩾3 LFC, while 11 DEGs were found to be upregulated ⩾3 LFC among the 445 DEGs identified in mock-inoculated samples. Genes related to the defense response, such as those in the MAPK signaling pathway, WRKY transcription factors, and other pathogenesis-related genes were identified among the highly expressed (⩾4 LFC) genes in the *C*Las-inoculated samples of rough lemon (Table 1). Upregulated genes greatly outnumbered downregulated genes in both *C*Las-inoculated samples of sweet orange and rough lemon (Figure 3). The number of downregulated DEGs was slightly higher than upregulated DEGs in mock-inoculated samples of sweet orange, while the number of upregulated DEGs was much greater than the number of downregulated DEGs in *C*Las-inoculated samples of sweet orange. A total of 29 and 25% of the DEGs in samples of *C*Las-inoculated rough lemon and sweet orange had a LFC⩾3, respectively, whereas only 1% of the DEGs in mock-inoculated samples of rough lemon and sweet orange were observed to have an LFC⩾3. These results indicate that overall gene transcription levels were higher in *C*Las-inoculated samples than in mock-inoculated samples of rough lemon and sweet orange.

**Figure 4:**
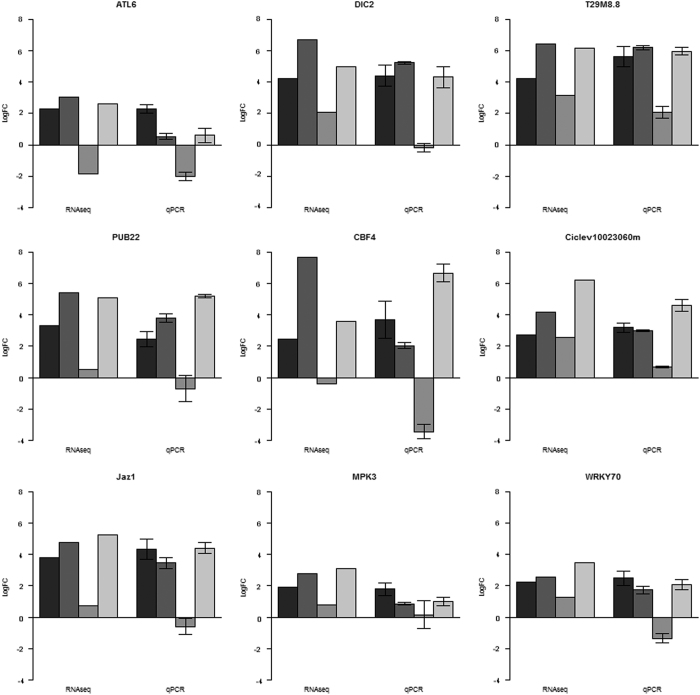
Relative expression of nine differentially expressed genes (DEGs) associated with disease resistance response as determined by RT-qPCR. Standard error bars are provided. *ATL6*: ubiquitin-protein ligase; *DIC2*: dicarboxylate carrier 2; *T29M8.8*: ethylene-responsive transcription factor; *PUB22*: ubiquitin-protein ligase PUB22; *CBF4*: DEHYDRATION-RESPO; Ciclev10023060m: hypothetical protein; *JAZ1*: JASMONATE-ZIM-DOMAIN PROTEIN; *MPK3*: MITOGEN-ACTIVATED PROTEIN KINASE 3; *WRKY70*: WRKY transcription factor 70.

Expression levels of 9 defense response-related genes were analyzed by RT-qPCR in order to confirm the results obtained by RNA-seq analysis. As shown in Figure 4, the relative levels of expression of the investigated genes were consistent between the RT-qPCR and RNA-seq data, with a few exceptions but within error range, indicating that the RNA-seq data were reliable.

### Functional categorization and subnetwork enrichment analysis

The Fisher Extraction Test and subnetwork enrichment analysis (SNEA), as provided in Blast2GO and Pathway Studio (Elsevier/Ariadne Genomics), were used to determine the specific Gene Ontology (GO) terms affected by *C*Las infection. GO terms associated with hormones, defense response, as well as stomatal and photosynthetic acclimation, were significantly over-represented based on the Fisher’s pairwise comparisons of *C*Las-inoculated and mock-inoculated genes in both rough lemon and sweet orange samples (Supplementary File 3, Supplementary Tables S1 and S2). Only 1 GO term (FDR⩽0.05), response to chitin, was identified in sweet orange, while 19 GO terms were identified in rough lemon samples (Table 2). Three of the top GO terms in rough lemon were response to chitin, ethylene biosynthetic process, and respiratory burst involved in defense response and response to molecules of bacterial origin (Table 2). Although a lower number of DEGs were identified in rough lemon than in sweet orange, more defense response GOs were associated with the transcriptomic response of rough lemon to *C*Las infection than in sweet orange, and may reflect the ‘tolerance’ response observed in rough lemon. The results of the Fisher’s Test indicated that ‘ATP binding’ was the most significantly enriched GO molecular function term in rough lemon, perhaps reflecting the significant number of genes under transcriptional regulation during the early response of rough lemon to *C*Las infection (Supplementary File 3, Supplementary Table S1). No statistically significant GO terms in the molecular function category were found in the sweet orange RNA-seq data (Supplementary File 3, Supplementary Table S2). SNEA uses a global expression regulatory network extracted from the entire PubMed database and full-text journals to extract regulatory networks. Using the non-parametric Mann-Whitney test, SNEA identified significant (*P*⩽0.05), over-represented (*P*⩽0.05) ontologies among the up- and downregulated DEGS that indicate an enrichment of each sub-network. A total of 32 and 44 ontologies associated with upregulated DEGs in *C*Las-inoculated rough lemon and sweet orange samples were identified, while 26 and 22 ontologies associated with downregulated DEGs were found in rough lemon and sweet orange, respectively (Supplementary File 4, Supplementary Tables S1 and S3). The top 3 ontologies for upregulated genes in *C*Las-inoculated samples of rough lemon were associated with defense response, biotic stress, and plant defense (Table 3a). In comparison, plant immunity, jasmonate response (JA), and shade avoidance were the top ontology terms identified in *C*Las-inoculated samples of sweet orange (Table 3b). Among downregulated DEGs, root phototropism, seed width, and cell expansion were the top 3 ontologies identified in *C*Las-inoculated samples of rough lemon; whereas root phototropism, response to ethylene stimulus and greening were the top ontologies identified in sweet orange (Tables 3a and b). In regards to mock-inoculated samples of rough lemon, photosynthesis, reductive pentose-phosphate cycle, and seed length were the top three ontologies identified for upregulated DEGs. On the other hand, plant response, nitrogen metabolism, and biotic stress were the top ontologies identified for downregulated DEGs. With respect to mock-inoculation of sweet orange, cuticle development, response to auxin stimulus, and ion homeostasis were the top ontologies identified for upregulated genes, whereas meristem development, meristem initiation, and leaf size were the top ontologies for downregulated genes (Supplementary File 4, Supplementary Tables S2 and S4).

### Transcription factors

A total of 68 transcription factors were identified (Table 4). Eighteen transcription factors (TFs) were identified in *C*Las-inoculated and mock-inoculated samples of rough lemon, all of which were related to plant immunity, and 12 of which had a transcription level ⩾3 LFC. Only one TF⩾3 LFC was observed in mock-inoculated rough lemon. ERF-1 was the only TF found in both *C*Las- and mock-inoculated samples of rough lemon, and was upregulated in *C*Las-inoculated samples and downregulated in mock-inoculated samples of rough lemon. A total of 34 TFs were identified in *C*Las-inoculated samples of sweet orange and 7 in mock-inoculated samples. *C*Las-inoculated samples of rough lemon had 15 upregulated versus 3 downregulated TFs, whereas 26 upregulated versus 10 down regulated TFs were identified in *C*Las-inoculated sweet orange. Only EIN3 was found to be downregulated in both *C*Las- and mock-inoculated sweet orange. Several TFs families, such as ATAF1, bHLH, ERF, MYC2, MYB, and WRKY, are specifically associated with regulating plant defense and immunity.^25^ A greater number of defense-related TFs were found in *C*Las-inoculated samples than in mock-inoculated samples. ERF-1, MYC2, RAP2.4, TFIID, and WRKY70 were associated with upregulation of jasmonate and ethylene metabolism. WRKY TFs exhibited stronger expression in *C*Las-inoculated rough lemon than in sweet orange (Table 4).

### Highly-expressed DEGs

DEGs involved in signal transduction were overwhelmingly observed and highly expressed in *C*Las-inoculated leaves of both rough lemon and sweet orange (Table 1). The *AT3G47570* gene, which encodes a protein that functions in protein phosphorylation in the transmembrane receptor protein tyrosine kinase signaling pathway, was the gene most hit (117 times) by citrus sequences obtained in the RNA-seq results. The R-gene, *AT2G20142* (TIR domain family protein), and *RBOHD* (respiration burst) were both more highly upregulated in rough lemon than in sweet orange. Two MAPK genes (*MPK3* and *MKK9*) were upregulated (LFC 3.1 and 1.51, respectively), in rough lemon, whereas only *MPK3* (LFC=2.79) was upregulated in sweet orange. *MAPKKK19*, *JAZ8*, *JAZ1*, *PUB22* (associated with respiratory burst involved in defense response), *DIC2* (ethylene biosynthetic process), *AT1G73805* (salicylic acid (SA) mediated signaling pathway), *CBF4* (abscisic acid signaling), *CYP94C1* (signal transduction), *MPK3*, WRKYs (*WRKY 40*, *WRKY 41*, *WRKY 46*, *WRKY 33*, *WRKY 70*), *ACS6*, *ERF-1* and *ERF9*, all of which are involved in signaling transduction pathway, were in the top 50 upregulated (LFC⩾3) DEGs in *C*Las-inoculated samples of rough lemon. The most highly upregulated (LFC⩾3) genes in *C*Las-inoculated sweet orange included a few DEGs involved in signaling pathways, such as *AT1G73805* (SAR-deficient 1), *CBF4*, *CYP94C1* and JAZ8, as well as other genes, such as *SRG3* (glycerol metabolic process), *F12M16.17* (transporter), *AT3G27640* (DNA dependent transporter), and *PP2-B15* (phloem protein) (Table 1, Supplementary File 2, Supplementary Table S3). The WRKY transcription factor, WRKY 41 (LFC=3.00), was ranked 89 among the most highly expressed DEGs *C*Las-inoculated sweet orange, while WRKY40 (LFC=4.94) was ranked 10 in the list of most highly upregulated genes in *C*Las-inoculated rough lemon. *CKI1* (cytokinin mediated signaling), *EBF1* (negative mediated ethylene signaling), *MJB24.15* (brassinosteroid stimulus), *PIL1* (red light signaling), *CYP71B37*, and *F4H6.15* (hypothetical protein) were among the most highly downregulated DEGs in *C*Las-inoculated rough lemon. *MMI9.1* (plant invertase/pectin methylesterase inhibitor), *UGE1* (UGE1 (UDP-D-glucose/UDP-D-galactose 4-epimerase 1), *AHP4* (cytokinin mediated signaling), *PRP2* (cell wall organization), and *AT3G62950* (cell redox homeostasis) were among the most highly downregulated DEGs in *C*Las-inoculated sweet orange.

## Discussion

The current study examined transcriptomic changes associated with HLB during the early stage of *C*Las infection in HLB-tolerant rough lemon and HLB-susceptible sweet orange. Detection of *C*Las in citrus is delayed after initial inoculation; trees remain visibly asymptomatic until the bacterial titer reaches levels sufficient for reliable detection by RT-qPCR.^17^ Infection of *C*Las-inoculated plants of rough lemon and sweet orange could not be confirmed by RT-qPCR in our study until 23 weeks after the plants had been inoculated. A previous study reported that HLB could be detected in sweet orange at 5–9 weeks after inoculation under greenhouse conditions.^10^ The discrepancy in the time needed for confirmation in the two studies is likely associated with the use of different inoculum sources with different titers of bacteria, and possibly differences in plant age and growing conditions. Stover *et al.*^26^ found that inoculation with Kuharske citrange results in lower *C*Las titers than other inoculum sources such as lemon and sweet oranges. Since both Kuharske and Carrizo are citranges, the inoculum used in our study could take a longer time to be detected by qPCR than ‘Lisbon’ lemon in the earlier study. Identifying the proper time point to collect samples to characterize an early response to infection can also be problematic. In our study, bulk samples were collected at 0, 5, 7, 9, 12 and 17 weeks in order to determine the appropriate time point to represent an early response. Preliminary RNA-seq data indicated that the number of DEGs changing in expression was maximized at week 7; as a result, this time point was selected for detailed sequencing and analysis (data not shown).

An appropriate statistical test is needed to determine whether or not observed differences in the number of obtained sequences (read counts) of a specific gene is significant, that is, whether the difference is greater than what would be expected due to natural random variation.^22^ In addition, it is also critical to design the experiment so that natural variation (not due to a treatment effect) in gene expression is minimized and the number of identified DEGs is maximized. Previous studies directly comparing *C*Las- and mock-inoculated plants, or tolerant and susceptible genotypes, may have not sufficiently reduced biological variation after inoculation at week 0. As a result, a low number of early-stage DEGs were identified.^4,10,11,27^ In the present study, gene expression in week 0 was used as a baseline in order to reduce the level of random variation between individual plants. To identify the DEGs induced by *C*Las, only DEGs that were uniquely expressed in *C*Las-inoculated and not in mock-inoculated samples at week 7 were selected for comparative analysis. Using this approach, we identified a greater number of DEGs in response to *C*Las inoculation than by direct pairwise comparisons of *C*Las-inoculated rough lemon and sweet orange at week 7. This approach has not been used in previous transcriptome studies of *C*Las infected citrus. However, we would not identify as DEGs those genes which had high absolute levels of expression in both week 0 and week 7 after inoculation. We speculate that some of these genes might play important roles against *C*Las, although these genes were not necessarily induced by *C*Las.

### Gene expression associated with tolerance and susceptibility to *C*Las

Plant disease resistance is generally divided into two categories, qualitative resistance, determined by major R-genes, and quantitative resistance, determined by multiple genes with minor effects.^28^ The first tier of defense, PTI, is triggered by the perception of PAMP/DAMPs by membrane-anchored PRRs, which is then followed by the activation of a MAPK cascade and downstream transcription factors, leading to immune responses. The second tier of defense is elicited by pathogen effectors via an interaction with an R protein (effector-triggered immunity), where the interaction between R protein and a pathogen effector oscillates between compatible and incompatible reactions over time. Plant pathogens are broadly divided into biotrophs and necrotrophs. Plant defense against biotrophic pathogens is largely due to major gene resistance.^29^ *C*Las is an obligate biotrophic pathogen and currently no resistant citrus varieties has been identified. R-gene-mediated resistance usually induces a hypersensitive response (HR), which is thought to combat biotrophic pathogens by restricting their access to water and nutrients.^29^ R gene-mediated resistance also activates SA-dependent signaling, leading to an activation of a string of presumed defense effector genes. The activation of SA signaling occurs throughout the plant to establish systemic acquired resistance (SAR) against subsequent pathogen infections.^29^ During SAR, deposition of callose and lignin occurs in plant cell walls, and plants acquire the ability to mount a rapid HR. Fan *et al.*^9^ reported that callose-plugged phloem sieve elements and inhibition of phloem loading were observed in *C*Las-inoculated leaves of both rough lemon and sweet orange, but that phloem transport was less affected in rough lemon than in sweet orange. In our study, phloem protein 2 (PP2–15) was identified as one of the most highly upregulated genes in *C*Las-infected sweet orange, but not in *C*Las-infected rough lemon. Several other studies also reported that phloem protein 2 (PP2) genes are upregulated in HLB-infected leaves.^4,9–11,14^ In addition to being involved in the differentiation of vascular tissue, a lectin-like protein (PP2) is associated with the plugging of sieve plates in response to wounding and as a defense against pathogens and insects.^30^ Accumulation of PP2 in sieve plates, in conjunction with phloem necrosis and blockage of the translocation stream, appears to be a major factor in disease symptom development of citrus greening.^11^ PP2-B15 was the most highly upregulated gene in *C*Las-inoculated roots of ‘Sanhu’ red tangerine at 50 days post inoculation. Zhong *et al.*^14^ speculated that PP2-like genes haves an active role in defense against the invading bacteria after a plant has been infected with *C*Las.

In our study, SNEA specifically identified ‘defense response’ (*P*=1.77E−06) and ‘plant immunity’ (*P*=0.0003) as over-represented ontologies among the upregulated genes (Table 3a and 3b) in *C*Las-inoculated leaves of rough lemon and sweet orange, respectively. Ontologies for ‘jasmonate response’ and ‘stomatal movement’ were also significantly over-represented in *C*Las-inoculated rough lemon. The accumulation of callose, which is synthesized between the cell wall and the plasma membrane, as well as stomatal closure, are classic markers of PTI.^31^ In addition, SA, JA, and ethylene hormones are induced during PTI. DEGs related to R gene-mediated resistance, which activates an SA-dependent signaling pathway, were not found in our study. *EDS1*and *PAD4,* which play important roles in SA signaling, and *NPR1*, a master regulator of SA,^32^ were also not significantly induced in any of the samples analyzed in the current study. Wang ^33,41^ indicated that *C*Las contains CLIBASIA_00255, which encodes a salicylate hydroxylase that can convert salicylic acid (SA) into catechol, a metabolite that does not induce a resistance response. ‘AT1G73805’, Systemic Acquired Resistance Deficient 1 (*SARD1,*), however, was highly expressed in *C*Las-inoculated samples of rough lemon (LFC=4.35) and sweet orange (LFC=9.00). *SARD1*, a plant-specific DNA-binding protein, is a key positive regulator of SA synthesis, and was induced after exposure to SA and JA.^34^ Knocking out *SARD1* compromises both basal resistance and SAR.^31^ Further study is needed to understand the mechanism of *SARD1* in citrus defense system. The SNEA analysis of the DEGs identified in the present study, specifically identified ‘hypersensitive response’ and ‘cell death’ as over-represented ontologies (*P*⩽0.05) among upregulated DEGs in *C*Las-inoculated leaves of both rough lemon and sweet orange, but these ontologies were not identified in mock-inoculated samples of rough lemon and sweet orange (Supplementary File 4). A total of 4 and 7 DEGs associated with hypersensitive response and cell death were identified in *C*Las-inoculated leaves of rough lemon, respectively, whereas 8 and 16 DEGs associated with hypersensitive response and cell death were identified in *C*Las-inoculated leaves of sweet orange, respectively. Among the identified DEGs, *BAP2*, *GLIP1* and *ACL5*, associated with inhibition of cell death, were found in *C*Las-inoculated samples of sweet orange, whereas only *BAP2* was found in *C*Las-inoculated samples of rough lemon. *Arabidopsis* C2 domain proteins, *BAP1*, and its homologue *BAP2*, negatively regulate biotic and abiotic cell death.^35^ *ACL5* controls *Arabidopsis* xylem morphogenesis through the prevention of premature cell death.^36^ Treatment of *Arabidopsis* plants with *GLIP1* protein systemically inhibited cell death in distant leaves inoculated with *A. brassicicola*, where cell death would be otherwise strongly induced.^37^ *MPK3*, *PLA2A*, *RBOHD*, *RPM1*, *SAUL1*, *SOBIR1*, *VTC2*, and *WRKY22*, all of which are associated with hypersensitive response and cell death, were upregulated in *C*Las-inoculated leaves of sweet orange, whereas *SOBIR1*, *MKK9*, *RBOHD*, and *MPK3,* also associated with hypersensitive response and cell death, were upregulated in *C*Las-inoculated leaves of rough lemon.

Starch, which accumulates extensively in photosynthetic cells, as well as in phloem elements and vascular parenchyma cells of leaf blades and petioles, has also been observed to accumulate in the xylem parenchyma and phelloderm of HLB-affected ‘Valencia’ orange trees but not in control (non-infected) samples.^38^ Albrecht and Bowman^4^ found that glucose-6-phosphate/phosphate transporter (*GPT2*), which mediates the import of glucose-6-phosphate, an essential substrate for starch biosynthesis, was more highly upregulated in infected ‘Cleopatra’ mandarin than in sweet orange. In the present study, *GPT2* expression was significantly induced to a high degree in infected rough lemon but not in sweet orange. A total of 19 enzymes belonging to the starch and sugar metabolism pathway were identified. Fourteen were identified in *C*Las-inoculated sweet orange and 5 in mock-inoculated sweet orange, whereas 6 sugar and starch metabolism enzymes were identified in mock-inoculated rough lemon and 2 in *C*Las-inoculated rough lemon. Synthase (ec:2.4.1.12), diphosphatase (ec:3.6.1.9), and glycogenase (ec:3.2.1.1) were also found to be upregulated in *C*Las-inoculated leaves of sweet orange, while saccharogen amylase (ec:3.2.1.2) was upregulated in mock-inoculated sweet orange. Synthases (ec:2.4.1.12, and ec:2.4.1.14) were upregulated in mock-inoculated rough lemon, while hexokinase type IV glucokinase (ec:2.7.1.1) and fructokinase (phosphorylating) (ec:2.7.1.4) were found in *C*las-inoculated samples of rough lemon (Supplementary File 5, Supplementary Tables S1 and S2). Although no symptoms of HLB were observed in inoculated leaves of rough lemon and sweet orange, a greater number of DEGs related to sugar and starch metabolism were found in inoculated leaves of sweet orange than in inoculated rough lemon leaves (Supplementary File 5, Supplementary Tables S1 and S2). These data indicate that transcriptomic reprogramming in carbohydrate metabolism and defense is occurring in response to *C*Las even before symptoms are visibly observed or positive RT-qPCR results for HLB were found. Previous studies have observed that many genes involved in photosynthesis are repressed in response to HLB, most likely due to increased accumulation of sucrose/glucose levels in leaves.^10,11,39^ In our study, SNEA analysis indicated that photosynthesis (*P*=0.001), as an ontology term, was the most significant in DEGs obtained from mock-inoculated rough lemon, whereas defense response (*P*=1.77 E−06), as an ontology term, was the most significant term among the DEGs obtained for *C*Las-inoculated rough lemon (Supplementary File 4, Supplementary Table S2). These data suggest that a dramatic shift from photosynthesis- to defense-related gene expression occurs in rough lemon as an early response to *C*Las. In previous proteome studies, it was found that accumulation of starch synthase increased, but the production of photosynthesis-related proteins decreased in infected rough lemon,^40^ and most significantly, upregulated proteins of infected sweet orange were involved in stress/defense response.^39^

### Regulation of WRKY expression by MAPKs

The peptide flg22, a bacterial flagella protein recognized by most plants, activates a MAPK cascade which then leads the activation of WRKY-type transcription factors, key regulators of plant defense.^41^ Flagella in *C*Las, however, have not been observed in any of the numerous electron micrographs of these bacteria infecting plants and psyllids.^2^ The reduced genome of *C*Las and their transmission by psyllids may allow it to avoid PTI. *C*Las, however, still possesses 57 genes coding for products that function in cell envelope biogenesis and the outer membrane, including lipopolysaccharides, and most flagellar genes, which might function as PAMPs.^33^ Elongation factor Tu (EF-Tu) is one of the most abundant bacterial proteins and is recognized as a PAMP by *Arabidopsis*.^42^ The plant PRR for EF-Tu is the LRR-RLK EF-Tu receptor (*EFR*), which belongs to the same subfamily (LRRXII) as *FLS2*.^42^ In this study, upregulation (LFC=2.84) of *EFR* was only found in inoculated sweet orange. No expression of *FLS2* was observed in *C*Las-inoculated leaves of either rough lemon or sweet orange.

**Figure 5:**
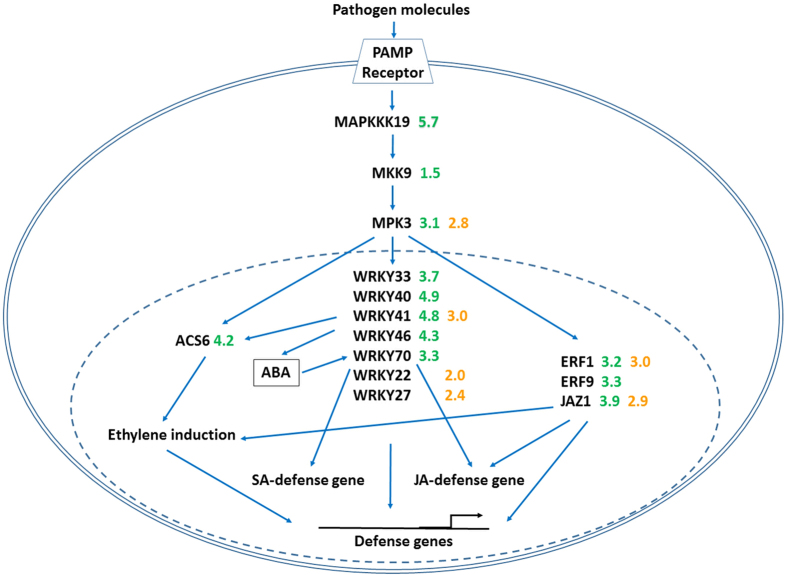
MAPK cascades as central signaling components in citrus pathogen defense. Upregulated genes in rough lemon are shown in green, and upregulated genes in sweet orange are shown in orange.

During PTI, activation of the MAPK cascade leads to the activation of WRKY-type transcription factors and other key regulators of plant immunity.^43^ It is thought that the MAPK cascade regulates plant immunity through the activation of defense-related genes via direct phosphorylation of downstream transcription factors, such as WRKYs and *ERF*s.^44,45^ *MKK9* is upstream of *MPK3*, and *MAPKKK19* is upstream of *MKK9* (Figure 5). In our study rough lemon exhibited a stronger MAPK response than sweet orange (Table 1). After the perception of flg22, *MPK6* activates ethylene biosynthesis through the phosphorylation of *ACS6*.^46^ *ACS6* was highly upregulated in *C*Las-inoculated rough lemon but not sweet orange. We also observed the strong upregulation of *ERF1* (LFC=3.18) and ERF9 (LFC=3.31) in *C*Las-inoculated leaves of rough lemon (Tables 3a and b, Figure 5). In response to pathogen attack, ET and JA cooperate through transcriptional induction of ET response factor 1 (*ERF1*).^47^ Activation of the MAPK cascade induces members of the WRKY family of transcription factors and defense-related genes ^48^ in tobacco.^49^ For example, *WRKY22* is activated via a MAPKcascade induced by flg22.^50^ In our study, *WRKY22* expression was only found in sweet orange, however, the upregulation was only moderate (LFC=1.96). These data were much lower than the upregulation of group 1 WRKY (*WRKY33*, *WRKY70* and *WRKY40*) TFs in rough lemon. Emerging evidence has indicated that group I WRKY transcription factors, which contain a conserved motif in the N-terminal region, are also activated by MAPK-dependent phosphorylation, underlining their importance in plant immunity.^51^

Our study indicates that WRKY transcription factor genes may play an important role in the tolerance to HLB exhibited by rough lemon. A total of eight WRKYs were identified in our RNA-seq analysis; with five in *C*Las-inoculated rough lemon and three in sweet orange. The magnitude of upregulation of all the WRKY genes in *C*Las-inoculated rough lemon was highly significant (LFC⩾3), while only *WRKY41* approached an increase of LFC=3.00 in *C*Las-inoculated sweet orange. Higashi *et al.*^52^ indicated that *WRKY41* may be a key regulator in the crosstalk between the salicylic acid and jasmonic acid pathways.^52^ In our study, *WRKY41* was upregulated in inoculated samples of both rough lemon and sweet orange. In response to mock-inoculation, however, only rough lemon exhibited downregulation (LFC=−1.62) of *WRKY42* and no significant difference was observed in sweet orange. The magnitude of the changes in the expression level of all of the WRKYs was higher in *C*Las-inoculated rough lemon than in *C*Las-inoculated sweet orange (Tables 3a and b). In *Arabidopsis*, *WRKY46* was specifically induced by salicylic acid (SA) and the biotrophic pathogen *Pseudomonas syringae*.^30,53^ The *WRKY33* transcription factor in *Arabidopsis* is required for resistance to necrotrophic fungal pathogens.^30^ Previous studies have presented evidence indicating that *WRKY70* regulates plant disease resistance,^53^ and identified *WRKY70* as a node of convergence for integrating salicylic acid (SA)- and jasmonic acid (JA)-mediated signaling events during plant response to bacterial pathogens.^30^ Over-expression of *WRKY70* in *Arabidopsis* resulted in the suppression of several JA responses, including expression of a subset of JA-responsive and *Alternaria brassicicola* responsive genes. SA-induced expression of *WRKY46*, *WRKY70,* and *WRKY53* is mainly dependent on *NPR1*.^30^ In our study, expression of *NPR1* did not respond significantly to either mock- or *C*Las-inoculation, however, *WRKY46* and *WRKY70* were strongly upregulated in *C*Las-inoculated rough lemon. Martinelli *et al.*^54^ reported that several WRKY members were more highly upregulated in fully ripe fruits than in young leaves of *C*las infected sweet orange.^54^

### ABA-induced activation of MAPKs in rough lemon

MAPK cascades (Figure 5) have also been shown to be involved in ABA signaling.^55^ In the present study, *MAK 3*, *MAPKKK19* and *MKK9* were all significantly upregulated in *C*Las-inoculated rough lemon (Supplementary Files, Supplementary Figures S1 and S2), and may play a role in the ABA signaling pathway as suggested in a previous *Arabidopsis* study. Blast2GO also indicated strong over representation of an ABA signaling GO term in *C*Las-inoculated rough lemon (Supplementary File 6). *MPK3* is activated by both H_2_O_2_ and ABA in *Arabidopsis* seedlings, and overexpression of *MPK3* increased ABA sensitivity to ABA-induced post-germination arrest of growth.^56^ *MPK9*, which is preferentially expressed in guard cells, is also activated by ABA and has been shown to mediate ABA signaling in guard cells.^57^ *MPK3/MPK6* negatively regulates ABA signaling in plants.^58^ The WRKY superfamily of TFs is the major regulator of plant defense and SA-mediated signaling, but also participates in ABA-mediated signaling.^59^ *AtWRKY40* has been reported to be a negative regulator of ABA signaling during seed germination and also interacts with *AtWRKY18* and *AtWRKY60* to inhibit the expression of stress-responsive genes.^53^ In our study, *WRKY40* was highly upregulated (LFC=4.9) in *C*Las-inoculated rough lemon. In addition, it was the most highly expressed among all of the upregulated WRKYs in *C*Las-inoculated rough lemon, but was not expressed in inoculated sweet orange. An and Mou^32^ stated that although ABA is known to play a crucial role in adaptation to abiotic stress, its role in biotic stress responses is less understood. In general, however, ABA is considered to be a negative regulator of disease resistance.^55^

Rapid production of reactive oxygen species (ROS) is one of the early detectable events following ABA perception.^60^ Plant recognition of PAMPs also induces the rapid and transient production of ROS in an oxidative burst.^55^ Interestingly, components of an ABA-activated MAPK cascade are also activated by ROS, suggesting that ABA and ROS may converge at the MAPK level in regulating stomatal closure.^55^ Previous studies of the hypersensitive response (HR) in pepper revealed that the activation of HR-induced cell death and defense responses is linked to signal transduction pathways that are coordinated by the activity of defense signaling molecules, such as ROS and plant defense hormones.^61^ In our current study, *PUB22* (respiration burst in defense) was highly upregulated in both *C*Las-inoculated rough lemon (LFC=5.08) and sweet orange (LFC=5.42). *RBOHD* (respiratory burst oxidase-D) was also upregulated in *C*Las-inoculated rough lemon (LFC=3.15) and sweet orange (LFC=2.88). ROS mediate ABA signaling in guard cells.^62^ Although most of the links between ABA and MAPKs are poorly understood, it is evident that these pathways are part of the complex cellular signaling network that exists in plants to integrate various environmental cues, such as pathogen challenges, nutrient status or developmental programs.^55^ *AtRbohD* and *AtRbohF* were initially described in *Arabidopsis* as key components of plant defense. *RBOH*-dependent ROS generation is associated with pathogen recognition during the oxidative burst linked to the perception of MAMPs/PAMPs and during the HR, which is coupled to the recognition of pathogen avirulence factors through resistance proteins.^37^

### Quantitative defense signaling

In a review, Eulgem^16^ stated that R-mediated resistance, basal defense, and SAR are related defense systems that share similar regulatory components and are effective against biotrophic pathogens. Differences between transcript profiles associated with R-mediated resistance and basal defense are quantitative rather than qualitative.^16^ Our results indicate that defense-response-related DEGs may be mediated by both basal defense and R-mediated resistance mechanisms. Distinct sets of DEGs were induced in *C*Las-inoculated rough lemon and sweet orange. We observed that the overall global mRNA expression (upregulated and downregulated) profiles in *C*Las-inoculated and mock-inoculated rough lemon and sweet orange were similar (Supplementary File 2, Supplementary Table S1). However, the amplitude of expression of the defense response in susceptible sweet orange was lower than in the tolerant rough lemon (Figure 3, Table 5). Resistance is regulated by changes in the levels of response of a variety of defense mechanisms and by quantitative enhancements that make the defense response more effective.^15,16,63^ The signaling mechanism that controls the activation of defense mechanisms consists of a highly interconnected network. Differences in the level of resistance often lie in the speed and intensity of specific defense responses. Resistant plants often respond more rapidly and vigorously to pathogens than susceptible plants. Hence, it is important to understand how plants sense the presence of a pathogen and initiate a subsequent response. Although the expression profile of up and downregulated genes in our study was similar in tolerant rough lemon and susceptible sweet orange, the proportion of downregulated DEGs with an LFC⩽−3 was 0.0% and 5.8% in rough lemon and sweet orange, respectively, while the proportion of upregulated DEGs with an LFC⩾3 was 29.0% and 25.1% in *C*Las-inoculated rough lemon and sweet orange, respectively. In mock-inoculated rough lemon and sweet orange, however, the proportion of DEGs that were down regulated was 3.8% and 1.2% in rough lemon and sweet orange, while the proportion of upregulated genes was 1.0% and 1.1%, respectively. RT-qPCR demonstrated that the transcription amplitude of 9 selected genes was higher in *C*Las-inoculated than in mock-inoculated treatments. In general, these data were consistent with the expression levels obtained from the RNA-seq data. The LFC values of *T29M8.8*, *AGP16*, *PUB22*, *CBF*, *AT1G32928*, *JAZ1*, *MPK3* and *WRKY70* were also higher in *C*Las-inoculated rough lemon than in the *C*Las-inoculated sweet orange. It should be noted, however, that there was a slight discrepancy between the expression levels of some genes in the RNA-seq data versus the RT-qPCR data, including *DIC2*, *EXL2*, and *HSP90.1.* Our results demonstrated that a higher number of DEGs with an LFC⩾3 were present in *C*Las-inoculated rough lemon sampled at week 7 post-inoculation. Activation of resistance and defense responses is stronger and faster in *C*Las-inoculated rough lemon than in *C*Las-inoculated sweet orange. Mock-inoculated plants of both rough lemon and sweet orange exhibited fewer WRKYs and defense-related genes than *C*Las-inoculated plants. These findings suggest that sweet orange exhibits an inability to suppress effectively the pathogen, resulting in the compatibility of the interaction between the host and pathogen. Maleck *et al.*^64^ found that defined sets of up-regulated genes are more highly expressed in an accelerated manner during incompatible interactions. Tao *et al.*^15^ proposed a mechanism that is commonly used in both R-gene and basal defense responses, where signal input to gene expression output occurs in a quantitatively determined manner. This mechanism is likely to involve regulators commonly used by both defense systems, such as WRKYs and defense response genes (Tables 4 and 5).

## Conclusions

The current study presents a comparative transcriptional analysis of the response of rough lemon and sweet orange to mock-inoculation and *C*Las-inoculation at 7 weeks post-inoculation in order to identify the early response to *C*Las infection in tolerant (rough lemon) and susceptible (sweet orange) trees. Rough lemon and sweet orange may recognize *C*Las via a pathogen recognition receptor, and activate plant immune systems even before positive RT-qPCR results can be obtained for CLas. Our collective results indicate that quantitative disease resistance may contribute to the durable tolerance to HLB exhibited by rough lemon, characterized by distinct transcriptional regulation of genes in various functional categories. Signaling pathways used by different defense systems appear to converge and target overlapping gene sets. The defense response DEGs mediated by basal defense and R-mediated resistance mechanisms were accelerated and amplified by *C*Las inoculation. Some transcription factors have been proven to play a pivotal role in disease resistance. Regulatory circuits linking signaling and gene regulation are emerging which suggest that a complex interplay between transcriptional activators and repressors fine-tunes the expression of the defense-related transcriptome. The current study provides new insights into the complex network of transcriptional regulation that occurs during the early response of rough lemon and sweet orange to *C*Las.

## Figures and Tables

**Table 1:** Differentially expressed genes (⩾4 log2 fold change) in rough lemon and sweet orange at week 7 compared to week 0

*Transcript name*	*Best-hit-arabi-name*	*Gene name*	*Fold*	*FDR*	*Description*
*Rough lemon inoculated*					
Ciclev10025910m	AT5G67080	*MAPKKK19*	5.71	0.000	Mitogen-activated protein kinase 19
Ciclev10010340m	AT2G46330	*AGP16*	5.41	0.000	Arabinogalactan protein 16
Ciclev10026376m	AT1G19180	*JAZ1*	5.26	0.001	Jasmonate-zim-domain protein 1
Ciclev10028254m	AT2G27690	*CYP94C1*	5.21	0.017	Cytochrome P450, family 94, subfamily C, polypeptide 1
Ciclev10017198m	AT1G30135	*JAZ8*	5.20	0.001	Jasmonate -zim-domain protein 8
Ciclev10011844m	AT3G52450	*PUB22*	5.09	0.018	Plant U-box 22
Ciclev10022337m	AT2G45760	*BAP2*	5.08	0.012	BON association protein 2
Ciclev10001822m	AT4G24570	*DIC2*	5.00	0.001	Dicarboxylate carrier 2
Ciclev10008930m	AT1G80840	*WRKY40*	4.94	0.000	WRKY D-binding protein 40
Ciclev10026527m	AT1G19180	*JAZ1*	4.93	0.009	Jasmonate-zim-domain protein 1
Ciclev10021038m	AT4G11070	*WRKY41*	4.76	0.001	WRKY family transcription factor
Ciclev10021738m	AT4G11070	*WRKY41*	4.75	0.001	WRKY family transcription factor
Ciclev10007843m	AT5G48150	*PAT1*	4.71	0.010	GRAS family transcription factor
Ciclev10017141m	Unknown		4.63	0.008	
Ciclev10022199m	AT4G11070	*WRKY41*	4.62	0.001	WRKY family transcription factor
Ciclev10028930m	AT4G34410	*RRTF1*	4.53	0.027	Redox responsive transcription factor 1
Ciclev10002273m	AT1G01720	*ATAF1*	4.49	0.007	NAC (No Apical Meristem) domain transcriptional regulator
Ciclev10002277m	AT1G01720	*ATAF1*	4.49	0.007	NAC (No Apical Meristem) domain transcriptional regulator
Ciclev10022487m	AT1G21010	*F9H16.19*	4.38	0.000	
Ciclev10028253m	AT2G27690	*CYP94C1*	4.38	0.025	Cytochrome P450, family 94, subfamily C, polypeptide 1
Ciclev10001956m	AT1G01720	*ATAF1*	4.37	0.008	NAC (No Apical Meristem) domain transcriptional regulator
Ciclev10019990m	AT1G73805	*AT1G73805*	4.35	0.000	Calmodulin binding protein-like
Ciclev10029464m	AT5G59820	*RHL41*	4.35	0.025	C2H2-type zinc finger family protein
Ciclev10020744m	AT2G46400	*WRKY46*	4.28	0.000	WRKY D-binding protein 46
Ciclev10019920m	AT4G11280	*ACS6*	4.24	0.000	1-Aminocyclopropane-1-carboxylic acid (acc) synthase 6
Ciclev10033110m	AT2G31945	*AT2G31945*	4.17	0.011	
Ciclev10015210m	AT1G61800	*GPT2*	4.15	0.011	Glucose-6-phosphate/phosphate translocator 2
Ciclev10015938m	AT1G61800	*GPT2*	4.13	0.012	Glucose-6-phosphate/phosphate translocator 2
Ciclev10015941m	AT1G61800	*GPT2*	4.13	0.012	Glucose-6-phosphate/phosphate translocator 2
Ciclev10005570m	AT5G51190	*MWD22.13*	4.11	0.000	Integrase-type D-binding superfamily protein
Ciclev10015511m	AT1G61800	*GPT2*	4.09	0.013	Glucose-6-phosphate/phosphate translocator 2
					
*Rough lemon mock-inoculated*					
Ciclev10017183m	AT4G15248	*AT4G15248*	4.79	0.043	B-box type zinc finger family protein
					
*Sweet orange inoculated*					
Ciclev10003623m	AT1G09155	*PP2-B15*	15.00	0.019	Phloem protein 2-B15
Ciclev10010533m	AT5G11330	*F2I11.220*	15.00	0.041	FAD/D(P)-binding oxidoreductase family protein
Ciclev10013424m	AT1G04360	*F19P19.21*	15.00	0.004	RING/U-box superfamily protein
Ciclev10013832m	AT5G38830	*K15E6.3*	15.00	0.039	Cysteinyl-tRNA synthetase, class Ia family protein
Ciclev10023904m	AT4G08850	*AT4G08850*	15.00	0.014	Leucine-rich repeat receptor-like protein kise family
Ciclev10024342m	AT1G73805	*AT1G73805*	15.00	0.009	Calmodulin binding protein-like
Ciclev10029883m	Unknown		15.00	0.044	
Ciclev10013766m	AT5G51990	*CBF4*	7.63	0.002	C-repeat-binding factor 4
Ciclev10015568m	AT3G02040	*SRG3*	7.41	0.000	senescence-related gene 3
Ciclev10010503m	AT1G53270	*F12M16.17*	7.32	0.003	ABC-2 type transporter family protein
Ciclev10014954m	AT3G27640	*AT3G27640*	7.31	0.017	Transducin/WD40 repeat-like superfamily protein
Ciclev10007406m	AT2G25600	*SPIK*	6.16	0.040	Shaker pollen inward K+ channel
Ciclev10028254m	AT2G27690	*CYP94C1*	6.07	0.003	Cytochrome P450, family 94, subfamily C, polypeptide 1
Ciclev10028253m	AT2G27690	*CYP94C1*	5.89	0.001	Cytochrome P450, family 94, subfamily C, polypeptide 1
Ciclev10013853m	AT3G47570	*AT3G47570*	5.44	0.006	Leucine-rich repeat protein kise family protein
Ciclev10011844m	AT3G52450	*PUB22*	5.42	0.007	Plant U-box 22
Ciclev10024282m	AT5G17680	*MVA3.30*	5.37	0.046	disease resistance protein (TIR-NBS-LRR class) family
Ciclev10026096m	AT1G17710	*F11A6.5*	5.30	0.008	Pyridoxal phosphate phosphatase-related protein
Ciclev10017198m	AT1G30135	*JAZ8*	5.21	0.001	Jasmonate-zim-domain protein 8
Ciclev10013223m	Unknown		5.18	0.047	
Ciclev10011408m	AT1G26810	*GALT1*	5.14	0.011	Galactosyltransferase1
Ciclev10032298m	AT4G00430	*PIP1;4*	5.09	0.000	Plasma membrane intrinsic protein 1;4
Ciclev10019961m	AT3G07250	*AT3G07250*	5.07	0.001	Nuclear transport factor 2 (NTF2) family protein
Ciclev10004868m	AT5G54010	*K19P17.18*	5.02	0.002	UDP-Glycosyltransferase superfamily protein
Ciclev10007591m	AT5G04860	*MUK11.18*	4.94	0.033	
Ciclev10013749m	AT1G68450	*T2E12.4*	4.94	0.023	VQ motif-containing protein
Ciclev10028305m	AT4G34131	*UGT73B3*	4.94	0.000	UDP-glucosyl transferase 73B3
Ciclev10032278m	AT5G05600	*MOP10.14*	4.92	0.013	2-Oxoglutarate and Fe(II)-dependent oxygese superf
Ciclev10031811m	AT5G05600	*MOP10.14*	4.81	0.007	2-Oxoglutarate and Fe(II)-dependent oxygese superf
Ciclev10031910m	AT5G05600	*MOP10.14*	4.79	0.009	2-Oxoglutarate and Fe(II)-dependent oxygese superf
Ciclev10017141m	Unknown		4.76	0.002	
Ciclev10021923m	AT4G25470	*CBF2*	4.76	0.016	C-Repeat/DRE binding factor 2
Ciclev10022337m	AT2G45760	*BAP2*	4.75	0.012	BON association protein 2
Ciclev10007652m	AT5G04860	*MUK11.18*	4.71	0.041	
Ciclev10008003m	AT5G04860	*MUK11.18*	4.67	0.038	
Ciclev10002675m	AT1G35210	*T32G9.25*	4.60	0.011	
Ciclev10031320m	AT2G44500	*F4I1.31*	4.59	0.001	O-fucosyltransferase family protein
Ciclev10013649m	AT4G26850	*VTC2*	4.57	0.000	mannose-1-phosphate guanylyltransferase
Ciclev10005385m	AT1G47990	*GA2OX4*	4.50	0.008	gibberellin 2-oxidase 4
Ciclev10023861m	AT4G28460	*F20O9.140*	4.48	0.013	
Ciclev10013959m	AT4G12010	*F16J13.80*	4.46	0.003	Disease resistance protein (TIR-NBS-LRR class) family
Ciclev10006910m	AT3G16520	*UGT88A1*	4.44	0.019	UDP-glucosyl transferase 88A1
Ciclev10005376m	AT5G49330	*MYB111*	4.44	0.019	myb domain protein 111
Ciclev10029690m	AT2G21650	*MEE3*	4.40	0.000	Homeodomain-like superfamily protein
Ciclev10023483m	AT4G01950	*GPAT3*	4.40	0.004	Glycerol-3-phosphate acyltransferase 3
Ciclev10022989m	AT1G03020	*F10O3.16*	4.36	0.044	Thioredoxin superfamily protein
Ciclev10010348m	AT1G19210	*T29M8.8*	4.33	0.016	Integrase-type D-binding superfamily protein
Ciclev10029663m	AT2G21650	*MEE3*	4.33	0.000	Homeodomain-like superfamily protein
Ciclev10000083m	AT1G75640	*F10A5.16*	4.32	0.000	Leucine-rich receptor-like protein kise family protein
Ciclev10020246m	AT4G00770	*T18A10.2*	4.26	0.019	
Ciclev10005777m	AT5G43650	*BHLH92*	4.23	0.018	Basic helix-loop-helix (bHLH) D-binding superfamily
Ciclev10032519m	AT5G13790	*AGL15*	4.23	0.002	AGAMOUS-like 15
Ciclev10029774m	Unknown		4.22	0.001	
Ciclev10030876m	AT1G10560	*PUB18*	4.18	0.000	Plant U-box 18
Ciclev10013961m	AT5G17680	*MVA3.30*	4.11	0.008	Disease resistance protein (TIR-NBS-LRR class) family
Ciclev10029578m	AT1G58420	*F9K23.5*	4.09	0.014	Uncharacterized conserved protein UCP031279
Ciclev10033986m	AT4G39670	*T19P19.60*	4.08	0.020	Glycolipid transfer protein (GLTP) family protein
Ciclev10022303m	AT5G52600	*MYB82*	4.03	0.000	myb domain protein 82
Ciclev10033777m	AT1G24430	*F21J9.9*	4.03	0.033	HXXXD-type acyl-transferase family protein
					
*Sweet orange mock-inoculated*					
Ciclev10029484m	UnKnown		4.74	0.033	
Ciclev10029585m	unknown		4.65	0.036	

Abbreviation: FDR, false discovery rate.

**Table 2:** Fisher's exact test with FDR (<0.05) between *C*Las- or inoculation and mock-inoculated rough lemon and sweet orange

*GO-ID*	*Term*	*FDR*
*CLas inoculated versus mock inoculated rough lemon*		
GO:0010200	Response to chitin	0.000
GO:0009693	Ethylene biosynthetic process	0.000
GO:0010288	Response to lead ion	0.000
GO:0002679	Respiratory burst involved in defense response	0.000
GO:0002237	Response to molecule of bacterial origin	0.010
GO:0009684	Indoleacetic acid biosynthetic process	0.013
GO:0010105	Negative regulation of ethylene-activated signaling pathway	0.016
GO:0009736	Cytokinin-activated signaling pathway	0.017
GO:0006569	Tryptophan catabolic process	0.024
GO:0050832	Defense response to fungus	0.029
GO:2000038	Regulation of stomatal complex development	0.040
GO:2000037	Regulation of stomatal complex patterning	0.040
GO:0080001	Mucilage extrusion from seed coat	0.040
GO:0048358	Mucilage pectin biosynthetic process	0.040
GO:0009690	Cytokinin metabolic process	0.040
GO:0009643	Photosynthetic acclimation	0.040
GO:0007276	Gamete generation	0.040
GO:0009401	Phosphoenolpyruvate-dependent sugar phosphotransferase system	0.040
		
*CLas inoculated versus mock inoculated sweet orange*		
GO:0010200	Response to chitin	0.039

Abbreviation: FDR, false discovery rate.

**Table 3:** Top 10 biological processes, as determined by sub-network enrichment analysis, associated with genes in rough lemon that were significantly (*P*<0.05) upregulated or downregulated in response to *C*Las infection

*Ontology*	*Gene name*	P*-value*
*Upregulated biological processes*		
Defense response	*RBOHD, MPK3, SOBIR1, WRKY70, RHL41, ATAF1, ERF-1, WRKY33, JAZ1, BAP2, WRKY40, STZ,BT1*	1.77E−06
Biotic stress trait	*ERF-1, WRKY33, MKK9, WRKY40, STZ*	1.30E−05
Plant defense	*RBOHD, MPK3, SOBIR1, WRKY70, ERF-1, NAC062, WRKY33, MKK9, WRKY40, STZ*	3.15E−05
Plant immunity	*MPK3, SOBIR1, ATAF1, ERF-1, NAC062, WRKY40*	5.92E−05
Leaf senescence	*SAUL1, WRKY70, ATAF1, JAZ8, MKK9, RAP2.4*	4.41E−04
Jasmonate response	*WRKY70, ERF-1, JAZ1, CML37*	5.24E−04
Stomatal movement	*RBOHD, MPK3, NCED3, CYP707A1, WRKY70, ATAF1, ERF-1, WRKY46*	5.30−04
Jasmonate metabolism	*WRKY70, ERF-1*	6.75E−04
Response to ethylene stimulus	*MPK3, ACS6, ERF-1, MKK9, RAP2.4*	7.33E−04
Abiotic stress	*MPK3, RHL41, ATAF1, NAC062, WRKY33, STZ*	8.21E−04
		
*Downregulated biological processes*		
Root phototropism	*NPH4, PKS1*	7.59E−05
Seed width	*NPH4, ARF8*	1.16E−04
Cell expansion	*ERS1, CKI1, NPH4, ARF8, TUB1*	1.33E−04
Hypocotyl growth	*CYP83B1, NPH4, PIL1, ARF8, PKS1*	1.88E−04
Hypocotyl shape	*NPH4, PKS1*	4.81E−04
Flower development	*ERS1, SEX1, DME, PIL1, ARF8, LUH*	1.81E−03
Fruit set	*NPH4, ARF8*	2.59E−03
Shade avoidance	*CYP83B1, PIL1*	2.69E−03
Response to ethylene stimulus	*ERS1, NPH4, EBF1*	3.17E−03
De-etiolation	*CYP83B1, PIL1*	3.92E−03

**Table 4:** Top 10 biological processes, as determined by sub-network enrichment analysis, associated with genes in sweet orange that were significantly (*P*<0.05) upregulated or downregulated in response to *C*Las infection

*Ontology*	*Gene name*	P*-value*
*Upregulated biological processes*		
Plant immunity	*GLIP1, MPK3, SOBIR1, EFR, ERF-1, NAC062, WRKY22, SNC4*	3.43E−04
Jasmonate response	*EFR, ERF-1, JAZ1, MYC2, ASK2, CML37*	3.60E−04
Shade avoidance	*COP1, PIF7, MYC2, BEE1*	1.20E−03
K^+^ Import/homeostasis	*RBOHD, SOS1, CHX20, ZIFL1*	1.59E−03
Cold acclimation	*MPK3, PPDK, COP1, RHL41, PHO1, CBF2, HSFA2,MYC2, CBF4*	1.69E−03
Disease resistance	*GLIP1,RBOHD,MPK3,SOBIR1,CCR1,VTC2,WIN2,PTF1,HSC70-1,HSP90.1,RPM1*	1.92E−03
Plant defense	*GLIP1,RBOHD,MPK3,SOBIR1,CCR1,FAB1,ERF-1,NAC062,MYC2,WRKY22,RPM1,YSL3,AAT1*	2.26E−03
Stomatal movement	*RBOHD,MPK3,PPDK,PUB18,COP1,NRT1.1,ERF-1,NHX2,HSC70-1,ALMT9,CHX20,ZIFL1*	3.01E−03
Floral organ abscission	*MPK3,HSL2,AGL15*	3.45E−03
Cryptochrome response	*COP1,SUB1*	4.18E−03
		
*Downregulated biological processes*		
Root phototropism	*GN,PKS1*	0.0006
Response to ethylene stimulus	*EFE,ERS1,EBF1,EIN3,ETR2*	0.0019
Greening	*PORA,EIN3,RR12,PKS1*	0.0030
Hypocotyl shape	*GN,PKS1*	0.0041
Vascularization	*ZLL,GN,RR12*	0.00427
Leaf size	*AVP1,TCTP,ETR2*	0.0054
Hypocotyl growth	*MUR4,CYP83B1,EIN3,FHY1,ETR2,PKS1*	0.00631
Gene silencing	*EFE,DME,ZLL,EMB25*	0.0071
Cation transport	*AVP1,CNGC1*	0.0076
Translation elongation	*MTO1,TCTP*	0.0076

**Table 5:** Fold-change of identified transcription factors

*Name*	*Mock inoculated rough lemon*	*CLas inoculated rough lemon*	*Mock inoculated sweet orange lemon*	*CLas inoculated sweet orange*
CBF4				7.6274
T29M8.8				4.3308
BHLH92				4.2331
MYB82				4.0349
NF-YA1				3.4703
EMB1444				3.2101
BEE1				3.1625
ERF-1		3.2017		3.0451
WRKY41		4.7115		3.0081
AT3G20640				2.9462
HSFA2				2.7241
WRKY27				2.4071
HSF4				2.3725
PIF7				1.9969
WRKY22				1.963
MYC2				1.8374
NF-YA7	1.3202			1.8007
F15H21.12		2.0221		1.7584
F6F3.7				1.7225
WLIM1				1.5062
T13M11.1				1.4698
TFIID				1.4263
TCP20				1.4007
ARF3				1.376
PTF1				1.3533
LRL1				1.1847
T4I9.13				−1.2194
EIN3	−1.4775		−1.2078	−1.3138
AT3G57795		−1.3328		−1.3829
AT1G05805				−1.5484
MUD21.1				−1.5583
MRH10.19	−2.1213			−1.6206
HMGB6				−1.738
MBK20.1				−2.6197
WRKY40		4.9433		
PAT1		4.7133		
RRTF1		4.5266		
ATAF1		4.4503		
WRKY46		4.2845		
MWD22.13		4.1084		
WRKY33		3.7237		
WRKY70		3.4712		
ERF9		3.3143		
ERF1	−1.9025	3.1845		
RAP2.4		2.282		
MUG13.28		1.2747		
GT2		−1.1792		
PIL1		−2.1031		
MYB66	3.2872			
F20P5.26	2.6354			
CGA1	2.3686			
HSFA6B	2.0545			
ILR3	1.877			
T6K21.80	1.7872			
NF-YC2	1.6739			
TTG2	1.5884			
UNE10	1.4733			
GBF3	1.4358			
YAB2	1.2666			
BZIP60	1.2192			
WRKY42	−1.6272			
AT3G10330	−2.3198			
AGL8			−3.5222	
AP2			1.2999	
AS1			1.6832	
HSFC1			−2.0526	
IAA8			1.6307	
T24P15.19			−1.8958	

**Table 6:** Fold-change of identified defense response-related genes

*Gene*	*Mock inoculated rough lemon*	*CLas inoculated rough lemon*	*Mock inoculated sweet orange lemon*	*CLas inoculated sweet orange*
*ATAF1*		4.5		
*ATL6*		−1.8		0.3
*BAP2*		4.2		4.8
*BT1*		2.2		
*CCR1*				2.8
*EFR*				
*ERF-1*				2.8
*GLIP1*		3.2		3.0
*JAZ1*				2.1
*MPK3*		3.9		2.9
*MYC2*		3.1		2.8
*RBOHD*				1.8
*RHL41*		3.2		2.9
*RPM1*		4.3		3.9
*SOBIR1*			2.1	1.9
*STZ*		2.9		2.6
*WRKY22*		3.3		
*WRKY33*				2.0
*WRKY40*		3.7		
*WRKY70*		4.9		
