# Supplementary material for: Reprogramming of a defense signaling pathway in rough lemon and sweet orange is a critical element of the early response to ‘Candidatus Liberibacter asiaticus’
Source: Hortic Res. 2017 Nov 29;4:17063–. doi: 10.1038/hortres.2017.63 (PMC5705785; doi:10.1038/hortres.2017.63)
Supplement: Additional File 6 [file hortres201763-s6.docx]

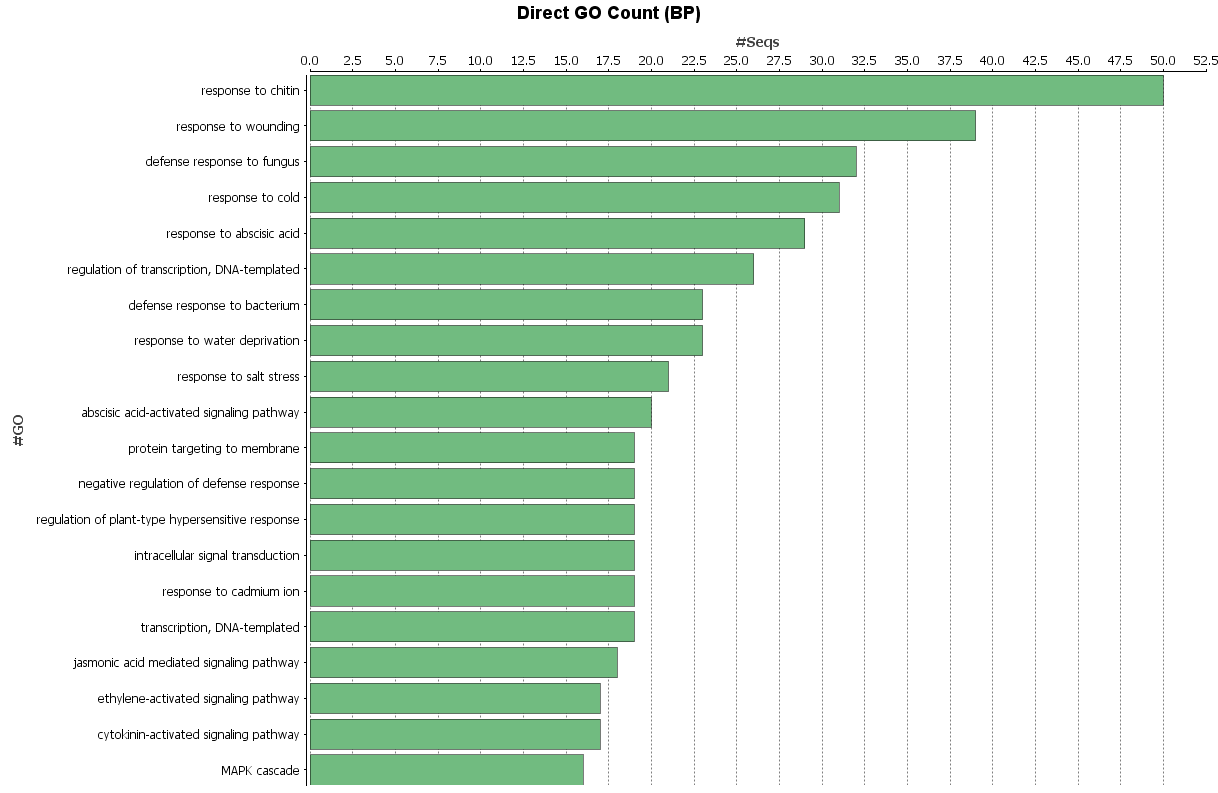


Figure S1: GO term for CLas-inoculated rough lemon


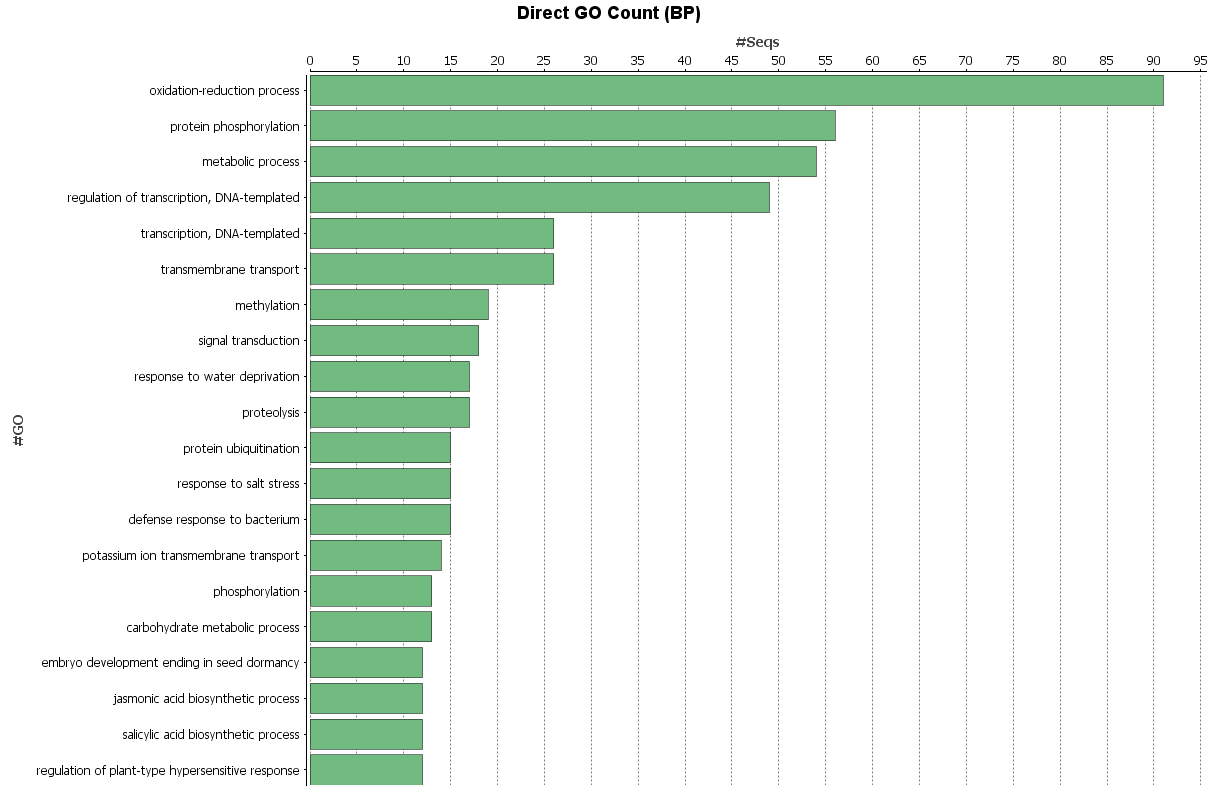


Figure S2: GO term for CLas-inoculated sweet orange

Table S1. Primers used for quantitative real time PCR.

| ID | type | sequence |  |
| --- | --- | --- | --- |
| Ciclev10001478m | Forward | GAGGAGGTGAAGGTAGTAGTAGAG | |
| Ciclev10001478m | Reverse | AAGCGATGATGCTCTTTGGA | |
| Ciclev10001822m | Forward | GGCTTTACGATGTGCTCAAAC | |
| Ciclev10001822m | Reverse | CAACTGCTCCGGAGATCAAA | |
| Ciclev10009540m | Forward | AGTACTTATCGCCGTCGATTTC | |
| Ciclev10009540m | Reverse | CCCATATTCGGAAGGGTAGTTATT | |
| Ciclev10011844m | Forward | GAGATGTTGCAGATTGGTGTTG | |
| Ciclev10011844m | Reverse | AGCACTTCCCTTGCCTTATC | |
| Ciclev10013766m | Forward | GCCGAACAAGAAGTCTCGAATA | |
| Ciclev10013766m | Reverse | AGAGTTTCCTCGTAAAGCCAAG | |
| Ciclev10023060m | Forward | CAAGATAACGCAGCAGAAACAG | |
| Ciclev10023060m | Reverse | TCAATATGGAACGATGGTCTCG | |
| Ciclev10026376m | Forward | TGCCTCTCTTCAACGTTTCTT | |
| Ciclev10026376m | Reverse | CCTGGATACTTGCCTTGATCTT | |
| Ciclev10028918m | Forward | GAAGCACTAGCCCATCCTTATC | |
| Ciclev10028918m | Reverse | TCTTCTCCCAGGCTTTGTTG | |
| Ciclev10032192m | Forward | TAGCATCCCGACAGAAGAAATC | |
| Ciclev10032192m | Reverse | CAATTGAATCCAGAGAGGACAAATC | |
|  |  |  | |
